# Cerebral Meningitis

**Published:** 1893-07

**Authors:** 


CEREBRAL MENINGITIS, by Martin W. Barr, M. D., Detroit, Mich., Geo. S. Davis.

One of "The Physicians Leisure Library," published by Geo. S. Davis, at $2.50 a year, or 25 cents a copy.

